# 1-(5-Nitro-2-oxoindolin-3-yl­idene)-4-*o*-tolyl­thio­semicarbazide methanol monosolvate

**DOI:** 10.1107/S1600536809043633

**Published:** 2009-10-28

**Authors:** Humayun Pervez, Muhammad Yaqub, Nazia Manzoor, M. Nawaz Tahir, M. Saeed Iqbal

**Affiliations:** a Department of Chemistry, Bahauddin Zakariya University, Multan 60800, Pakistan; b Department of Physics, University of Sargodha, Sargodha, Pakistan; c Department of Chemistry, Government College University, Lahore, Pakistan

## Abstract

In the title compound, C_16_H_13_N_5_O_3_S·CH_4_O, the dihedral angle between the isatin unit and the 2-methyl­phenyl group is 41.81 (2)° and intra­molecular N—H⋯O and N—H⋯N hydrogen bonds occur, generating *S*(6) and *S*(5) rings, respectively. In the crystal, polymeric chains arise as a result of N—H⋯O, O—H⋯S and C—H⋯O inter­actions.

## Related literature

For related structures, see: Revenko *et al.* (1994 ▶); Pervez *et al.* (2009 ▶). For graph-set theory, see: Bernstein *et al.* (1995 ▶).
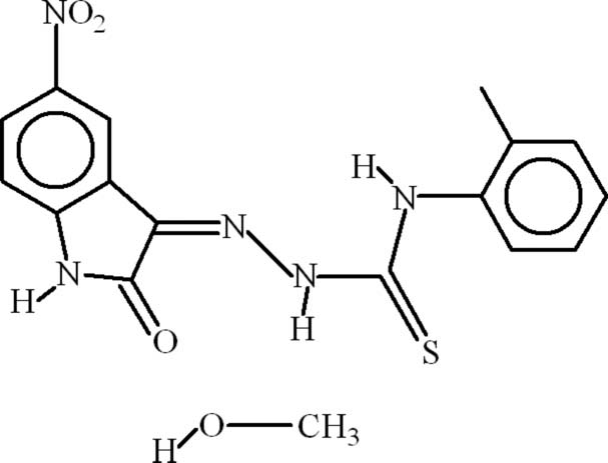


## Experimental

### 

#### Crystal data

C_16_H_13_N_5_O_3_S·CH_4_O
*M* *_r_* = 387.42
Monoclinic, 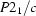

*a* = 14.2485 (5) Å
*b* = 7.6986 (3) Å
*c* = 18.5937 (6) Å
β = 119.847 (2)°
*V* = 1769.07 (11) Å^3^
*Z* = 4
Mo *K*α radiation
μ = 0.22 mm^−1^
*T* = 296 K
0.30 × 0.16 × 0.12 mm

#### Data collection

Bruker Kappa APEXII CCD diffractometer
Absorption correction: multi-scan (*SADABS*; Bruker, 2005 ▶) *T*_min_ = 0.963, *T*_max_ = 0.974
18543 measured reflections
4005 independent reflections
2975 reflections with *I* > 2σ(*I*)
*R*_int_ = 0.031

#### Refinement

*R*[*F* ^2^ > 2σ(*F* ^2^)] = 0.038
*wR*(*F* ^2^) = 0.098
*S* = 1.03
4005 reflections
249 parameters
H-atom parameters constrained
Δρ_max_ = 0.24 e Å^−3^
Δρ_min_ = −0.22 e Å^−3^

### 

Data collection: *APEX2* (Bruker, 2007 ▶); cell refinement: *SAINT* (Bruker, 2007 ▶); data reduction: *SAINT*; program(s) used to solve structure: *SHELXS97* (Sheldrick, 2008 ▶); program(s) used to refine structure: *SHELXL97* (Sheldrick, 2008 ▶); molecular graphics: *ORTEP-3 for Windows* (Farrugia, 1997 ▶) and *PLATON* (Spek, 2009 ▶); software used to prepare material for publication: *WinGX* (Farrugia, 1999 ▶) and *PLATON*.

## Supplementary Material

Crystal structure: contains datablocks global, I. DOI: 10.1107/S1600536809043633/hb5167sup1.cif

Structure factors: contains datablocks I. DOI: 10.1107/S1600536809043633/hb5167Isup2.hkl

Additional supplementary materials:  crystallographic information; 3D view; checkCIF report

## Figures and Tables

**Table 1:** Hydrogen-bond geometry (Å, °)

*D*—H⋯*A*	*D*—H	H⋯*A*	*D*⋯*A*	*D*—H⋯*A*
N3—H3⋯O1	0.86	2.04	2.7074 (17)	134
N4—H4*A*⋯N2	0.86	2.20	2.6254 (18)	110
N1—H1⋯O4^i^	0.86	2.02	2.8394 (19)	160
O4—H4*B*⋯S1^ii^	0.82	2.55	3.3485 (14)	164
C16—H16*C*⋯O3^iii^	0.96	2.45	3.342 (3)	154

Symmetry codes: (i) 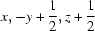; (ii) 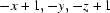; (iii) 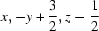.

**Fig. 1.**
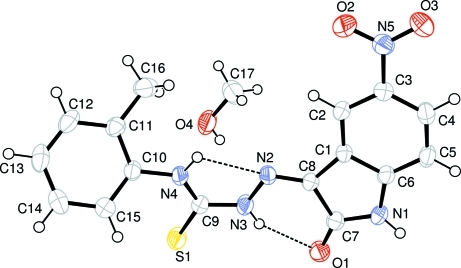
View of (I) with displacement ellipsoids drawn at the 50% probability level. H-atoms are shown by circles of arbitrary radius and the dotted lines represent the intramolecular H-bonds.

**Fig. 2.**
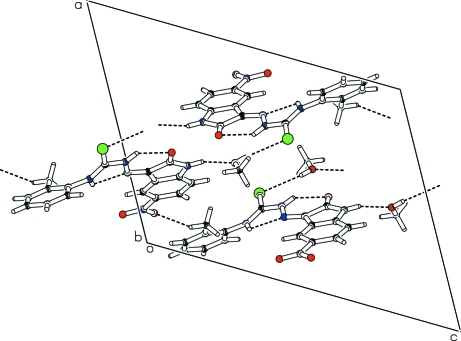
The partial packing of (I), which shows that molecules form infinite one dimensional polymeric chains.

## supplementary crystallographic information

### Comment

Recently we have reported the preparation and crystal structure of (*Z*)-4-Hexyl-1-(5-nitro-2-oxo-2,3-dihydro-1H-indol-3-ylidene) thiosemicarbazide (Pervez *et al.*, 2009). The title compound (I, Fig. 1) has been prepared and being reported in continuation of synthesizing various isatin derivatives due to their importance.

The crystal structure of (II) Isatin β-4-(p-tolyl)thiosemicarbazone (Revenko *et al.*, 1994) has been published. The title compound (I) differs from (II) due to attachment of NO_2_ group with isatin and positional change of CH_3_ group on the benzene ring.

In the crystal structure of (I), the group A (C1—C8/N1/N2/N3/O1) of isatin moiety and 2-methylphenyl group B (C10–C17) are planar with a maximum r. m. s. deviations of  0.0187  and 0.0065 Å respectively, from their mean square plane. The dihedral angle between A/B is 41.81 (2)°. The nitro group C (N2/O2/O3) is oriented at a dihedral angle of 5.7 (2)° with group A. In (I), there exist two interamolecular H-bondings resulting in S(5) and S(6) ring motifs (Bernstein *et al.*, 1995). Methanol monosolvate interlinks the molecules through H-bondings (Table 1., Fig. 2). The molecules are stabilized in the form of infinite one dimensional polymeric chains.

### Experimental

4-*o*-Tolylthiosemicarbazide (0.45 g, 2.5 mmol) dissolved in ethanol (10 ml) was added to a hot solution of 5-nitroisatin (0.46 g, 2.5 mmol) in 50% aqueous ethanol (30 ml) containing a few drops of glacial acetic acid. The reaction mixture was then refluxed for 2 h. The yellow crystalline solid formed during heating under reflux was collected by suction filtration. Thorough washing with hot aqueous ethanol furnished the title compound (I) in pure form (0.71 g, 80%), m.p. 499 K. The yellow needles of (I) were grown in ethanol:n-hexane (1:4) system at room temperature by diffusion method.

### Refinement

The H-atoms were positioned geometrically (O–H = 0.82 Å, N–H = 0.86 Å, C–H = 0.93–0.96 Å) and refined as riding with *U*_iso_(H) = 1.2*U*_eq_(carrier) or 1.5*U*_eq_(methyl C).

### Crystal data

**Table:**

C_16_H_13_N_5_O_3_S·CH_4_O	*F*(000) = 808
*M**_r_* = 387.42	*D*_x_ = 1.455 Mg m^−^^3^
Monoclinic, *P*2_1_/*c*	Mo *K*α radiation, λ = 0.71073 Å
Hall symbol: -P 2ybc	Cell parameters from 2975 reflections
*a* = 14.2485 (5) Å	θ = 2.5–27.5°
*b* = 7.6986 (3) Å	µ = 0.22 mm^−^^1^
*c* = 18.5937 (6) Å	*T* = 296 K
β = 119.847 (2)°	Cut needle, yellow
*V* = 1769.07 (11) Å^3^	0.30 × 0.16 × 0.12 mm
*Z* = 4	

### Data collection

**Table:**

Bruker Kappa APEXII CCD diffractometer	4005 independent reflections
Radiation source: fine-focus sealed tube	2975 reflections with *I* > 2σ(*I*)
graphite	*R*_int_ = 0.031
Detector resolution: 7.60 pixels mm^-1^	θ_max_ = 27.5°, θ_min_ = 2.5°
ω scans	*h* = −18→18
Absorption correction: multi-scan (*SADABS*; Bruker, 2005)	*k* = −10→10
*T*_min_ = 0.963, *T*_max_ = 0.974	*l* = −23→23
18543 measured reflections	

### Refinement

**Table:**

Refinement on *F*^2^	Primary atom site location: structure-invariant direct methods
Least-squares matrix: full	Secondary atom site location: difference Fourier map
*R*[*F*^2^ > 2σ(*F*^2^)] = 0.038	Hydrogen site location: inferred from neighbouring sites
*wR*(*F*^2^) = 0.098	H-atom parameters constrained
*S* = 1.03	*w* = 1/[σ^2^(*F*_o_^2^) + (0.0413*P*)^2^ + 0.4905*P*] where *P* = (*F*_o_^2^ + 2*F*_c_^2^)/3
4005 reflections	(Δ/σ)_max_ < 0.001
249 parameters	Δρ_max_ = 0.24 e Å^−^^3^
0 restraints	Δρ_min_ = −0.22 e Å^−^^3^

### Special details

**Table:**

Geometry. Bond distances, angles *etc*. have been calculated using the rounded fractional coordinates. All su's are estimated from the variances of the (full) variance-covariance matrix. The cell e.s.d.'s are taken into account in the estimation of distances, angles and torsion angles
Refinement. Refinement of *F*^2^ against ALL reflections. The weighted *R*-factor *wR* and goodness of fit *S* are based on *F*^2^, conventional *R*-factors *R* are based on *F*, with *F* set to zero for negative *F*^2^. The threshold expression of *F*^2^ > σ(*F*^2^) is used only for calculating *R*-factors(gt) *etc*. and is not relevant to the choice of reflections for refinement. *R*-factors based on *F*^2^ are statistically about twice as large as those based on *F*, and *R*- factors based on ALL data will be even larger.

### Fractional atomic coordinates and isotropic or equivalent isotropic displacement parameters (Å^2^)

**Table:**

	*x*	*y*	*z*	*U*_iso_*/*U*_eq_	
S1	0.36128 (4)	−0.18526 (6)	0.42815 (3)	0.0480 (2)	
O1	0.43184 (10)	0.09444 (15)	0.66176 (7)	0.0471 (4)	
O2	0.09516 (11)	0.88792 (17)	0.44105 (8)	0.0535 (5)	
O3	0.14063 (12)	1.04229 (17)	0.54995 (9)	0.0631 (5)	
N1	0.39348 (11)	0.35294 (18)	0.70502 (8)	0.0388 (4)	
N2	0.28711 (10)	0.24879 (17)	0.49325 (8)	0.0342 (4)	
N3	0.33161 (11)	0.09298 (17)	0.49406 (8)	0.0390 (4)	
N4	0.20990 (10)	0.06337 (17)	0.35699 (8)	0.0364 (4)	
N5	0.14422 (11)	0.90750 (18)	0.51621 (9)	0.0408 (5)	
C1	0.28486 (11)	0.4853 (2)	0.58087 (9)	0.0309 (4)	
C2	0.22200 (12)	0.6183 (2)	0.52973 (10)	0.0329 (5)	
C3	0.21086 (12)	0.7648 (2)	0.56800 (10)	0.0342 (5)	
C4	0.25938 (13)	0.7812 (2)	0.65330 (10)	0.0380 (5)	
C5	0.32173 (13)	0.6488 (2)	0.70422 (10)	0.0385 (5)	
C6	0.33402 (12)	0.5016 (2)	0.66721 (9)	0.0330 (4)	
C7	0.38889 (12)	0.2370 (2)	0.64829 (10)	0.0362 (5)	
C8	0.31690 (12)	0.3173 (2)	0.56487 (9)	0.0314 (4)	
C9	0.29577 (12)	−0.0024 (2)	0.42330 (9)	0.0346 (5)	
C10	0.14634 (12)	−0.0135 (2)	0.27704 (9)	0.0330 (5)	
C11	0.11584 (12)	0.0923 (2)	0.20843 (10)	0.0367 (5)	
C12	0.04962 (14)	0.0193 (3)	0.13070 (10)	0.0470 (6)	
C13	0.01474 (14)	−0.1498 (3)	0.12170 (11)	0.0501 (6)	
C14	0.04524 (13)	−0.2512 (2)	0.19058 (12)	0.0466 (6)	
C15	0.11076 (14)	−0.1830 (2)	0.26893 (11)	0.0413 (5)	
C16	0.15262 (16)	0.2771 (3)	0.21779 (12)	0.0543 (6)	
O4	0.46564 (14)	0.2556 (2)	0.37038 (8)	0.0764 (6)	
C17	0.43827 (17)	0.4240 (3)	0.37829 (13)	0.0599 (7)	
H1	0.42879	0.33638	0.75775	0.0465*	
H2	0.18875	0.60978	0.47231	0.0395*	
H3	0.38341	0.05337	0.53999	0.0468*	
H4	0.24971	0.88234	0.67621	0.0456*	
H4A	0.19014	0.16531	0.36307	0.0437*	
H5	0.35450	0.65800	0.76159	0.0462*	
H12	0.02844	0.08685	0.08361	0.0563*	
H13	−0.02945	−0.19550	0.06905	0.0601*	
H14	0.02187	−0.36585	0.18459	0.0559*	
H15	0.13064	−0.25081	0.31575	0.0496*	
H16A	0.23026	0.28089	0.24674	0.0814*	
H16B	0.12630	0.34033	0.24866	0.0814*	
H16C	0.12490	0.32864	0.16396	0.0814*	
H4B	0.50868	0.21697	0.41631	0.0916*	
H17A	0.38390	0.42215	0.39442	0.0898*	
H17B	0.41066	0.48319	0.32618	0.0898*	
H17C	0.50125	0.48363	0.41975	0.0898*	

### Atomic displacement parameters (Å^2^)

**Table:**

	*U*^11^	*U*^22^	*U*^33^	*U*^12^	*U*^13^	*U*^23^
S1	0.0570 (3)	0.0429 (3)	0.0355 (3)	0.0174 (2)	0.0165 (2)	−0.0014 (2)
O1	0.0506 (7)	0.0385 (7)	0.0345 (7)	0.0088 (5)	0.0077 (5)	0.0026 (5)
O2	0.0627 (8)	0.0480 (8)	0.0382 (8)	0.0117 (6)	0.0164 (6)	0.0063 (6)
O3	0.0910 (10)	0.0387 (8)	0.0612 (9)	0.0172 (7)	0.0390 (8)	−0.0016 (7)
N1	0.0443 (7)	0.0392 (8)	0.0220 (7)	−0.0009 (6)	0.0083 (6)	−0.0004 (6)
N2	0.0372 (7)	0.0302 (7)	0.0291 (7)	0.0014 (5)	0.0119 (6)	−0.0027 (6)
N3	0.0428 (7)	0.0361 (8)	0.0265 (7)	0.0084 (6)	0.0084 (6)	−0.0021 (6)
N4	0.0441 (7)	0.0288 (7)	0.0285 (7)	0.0046 (6)	0.0121 (6)	−0.0030 (6)
N5	0.0475 (8)	0.0347 (8)	0.0436 (9)	0.0026 (6)	0.0253 (7)	0.0013 (7)
C1	0.0333 (7)	0.0308 (8)	0.0254 (8)	−0.0043 (6)	0.0122 (6)	−0.0032 (6)
C2	0.0359 (8)	0.0346 (8)	0.0251 (8)	−0.0021 (6)	0.0129 (6)	−0.0012 (7)
C3	0.0361 (8)	0.0322 (8)	0.0341 (9)	−0.0005 (6)	0.0174 (7)	0.0012 (7)
C4	0.0428 (8)	0.0359 (9)	0.0364 (9)	−0.0039 (7)	0.0205 (7)	−0.0094 (7)
C5	0.0430 (9)	0.0437 (10)	0.0258 (8)	−0.0063 (7)	0.0149 (7)	−0.0077 (7)
C6	0.0346 (7)	0.0333 (8)	0.0269 (8)	−0.0052 (6)	0.0122 (6)	−0.0016 (7)
C7	0.0355 (8)	0.0348 (9)	0.0285 (8)	−0.0031 (7)	0.0085 (6)	−0.0006 (7)
C8	0.0325 (7)	0.0305 (8)	0.0254 (8)	−0.0024 (6)	0.0100 (6)	−0.0006 (7)
C9	0.0397 (8)	0.0339 (9)	0.0288 (8)	0.0004 (7)	0.0159 (7)	−0.0005 (7)
C10	0.0339 (7)	0.0347 (9)	0.0278 (8)	0.0030 (6)	0.0134 (6)	−0.0027 (7)
C11	0.0347 (8)	0.0391 (9)	0.0333 (9)	0.0045 (7)	0.0146 (7)	0.0024 (7)
C12	0.0433 (9)	0.0588 (12)	0.0279 (9)	0.0085 (8)	0.0095 (7)	0.0049 (8)
C13	0.0396 (9)	0.0605 (12)	0.0356 (10)	0.0019 (8)	0.0077 (8)	−0.0153 (9)
C14	0.0428 (9)	0.0401 (10)	0.0518 (11)	−0.0056 (8)	0.0196 (8)	−0.0132 (9)
C15	0.0472 (9)	0.0368 (9)	0.0390 (10)	−0.0003 (7)	0.0207 (8)	0.0001 (8)
C16	0.0581 (11)	0.0461 (11)	0.0468 (11)	−0.0016 (9)	0.0171 (9)	0.0114 (9)
O4	0.1094 (13)	0.0594 (9)	0.0329 (8)	0.0304 (9)	0.0147 (8)	−0.0028 (7)
C17	0.0589 (11)	0.0478 (12)	0.0609 (14)	0.0006 (9)	0.0206 (10)	−0.0080 (10)

### Geometric parameters (Å, °)

**Table:**

S1—C9	1.6666 (17)	C5—C6	1.381 (2)
O1—C7	1.220 (2)	C7—C8	1.502 (2)
O2—N5	1.2215 (19)	C10—C11	1.389 (2)
O3—N5	1.227 (2)	C10—C15	1.381 (2)
O4—C17	1.383 (3)	C11—C16	1.496 (3)
O4—H4B	0.8200	C11—C12	1.392 (2)
N1—C7	1.358 (2)	C12—C13	1.374 (3)
N1—C6	1.389 (2)	C13—C14	1.373 (3)
N2—N3	1.353 (2)	C14—C15	1.384 (3)
N2—C8	1.292 (2)	C2—H2	0.9300
N3—C9	1.365 (2)	C4—H4	0.9300
N4—C10	1.428 (2)	C5—H5	0.9300
N4—C9	1.331 (2)	C12—H12	0.9300
N5—C3	1.458 (2)	C13—H13	0.9300
N1—H1	0.8600	C14—H14	0.9300
N3—H3	0.8600	C15—H15	0.9300
N4—H4A	0.8600	C16—H16C	0.9600
C1—C2	1.381 (2)	C16—H16A	0.9600
C1—C8	1.451 (2)	C16—H16B	0.9600
C1—C6	1.402 (2)	C17—H17A	0.9600
C2—C3	1.384 (2)	C17—H17B	0.9600
C3—C4	1.385 (2)	C17—H17C	0.9600
C4—C5	1.374 (2)		
			
C17—O4—H4B	109.00	N4—C10—C15	120.89 (14)
C6—N1—C7	111.53 (13)	N4—C10—C11	117.36 (14)
N3—N2—C8	116.02 (13)	C10—C11—C12	117.23 (16)
N2—N3—C9	121.18 (13)	C10—C11—C16	121.31 (15)
C9—N4—C10	128.37 (14)	C12—C11—C16	121.46 (16)
O2—N5—C3	118.45 (14)	C11—C12—C13	121.76 (17)
O3—N5—C3	118.61 (14)	C12—C13—C14	119.86 (17)
O2—N5—O3	122.94 (15)	C13—C14—C15	120.05 (16)
C6—N1—H1	124.00	C10—C15—C14	119.48 (15)
C7—N1—H1	124.00	C1—C2—H2	122.00
N2—N3—H3	119.00	C3—C2—H2	122.00
C9—N3—H3	119.00	C5—C4—H4	120.00
C10—N4—H4A	116.00	C3—C4—H4	120.00
C9—N4—H4A	116.00	C4—C5—H5	121.00
C2—C1—C6	120.36 (15)	C6—C5—H5	121.00
C2—C1—C8	133.02 (14)	C13—C12—H12	119.00
C6—C1—C8	106.61 (13)	C11—C12—H12	119.00
C1—C2—C3	116.82 (15)	C12—C13—H13	120.00
N5—C3—C4	118.52 (14)	C14—C13—H13	120.00
N5—C3—C2	118.56 (14)	C15—C14—H14	120.00
C2—C3—C4	122.92 (15)	C13—C14—H14	120.00
C3—C4—C5	120.31 (15)	C10—C15—H15	120.00
C4—C5—C6	117.66 (15)	C14—C15—H15	120.00
N1—C6—C1	109.71 (14)	C11—C16—H16B	109.00
N1—C6—C5	128.35 (14)	C11—C16—H16C	109.00
C1—C6—C5	121.94 (15)	C11—C16—H16A	109.00
N1—C7—C8	106.00 (13)	H16A—C16—H16C	109.00
O1—C7—C8	126.66 (15)	H16B—C16—H16C	109.00
O1—C7—N1	127.32 (15)	H16A—C16—H16B	109.00
N2—C8—C1	126.86 (14)	O4—C17—H17A	109.00
C1—C8—C7	106.14 (13)	O4—C17—H17B	109.00
N2—C8—C7	126.98 (15)	O4—C17—H17C	109.00
N3—C9—N4	114.76 (14)	H17A—C17—H17B	109.00
S1—C9—N4	127.11 (12)	H17A—C17—H17C	109.00
S1—C9—N3	118.14 (12)	H17B—C17—H17C	109.00
C11—C10—C15	121.60 (15)		
			
C7—N1—C6—C1	1.2 (2)	C6—C1—C8—N2	178.05 (18)
C7—N1—C6—C5	−178.24 (19)	C6—C1—C8—C7	−0.3 (2)
C6—N1—C7—O1	−179.54 (19)	C1—C2—C3—N5	−179.37 (17)
C6—N1—C7—C8	−1.4 (2)	C1—C2—C3—C4	0.0 (3)
C8—N2—N3—C9	171.16 (17)	N5—C3—C4—C5	179.22 (18)
N3—N2—C8—C1	177.28 (17)	C2—C3—C4—C5	−0.2 (3)
N3—N2—C8—C7	−4.8 (3)	C3—C4—C5—C6	0.3 (3)
N2—N3—C9—S1	175.23 (13)	C4—C5—C6—N1	179.14 (19)
N2—N3—C9—N4	−5.1 (2)	C4—C5—C6—C1	−0.3 (3)
C10—N4—C9—S1	7.3 (3)	O1—C7—C8—N2	0.9 (3)
C10—N4—C9—N3	−172.36 (17)	O1—C7—C8—C1	179.19 (19)
C9—N4—C10—C11	−137.33 (19)	N1—C7—C8—N2	−177.33 (18)
C9—N4—C10—C15	47.0 (3)	N1—C7—C8—C1	1.0 (2)
O2—N5—C3—C2	4.6 (3)	N4—C10—C11—C12	−177.02 (18)
O2—N5—C3—C4	−174.76 (18)	N4—C10—C11—C16	2.9 (3)
O3—N5—C3—C2	−174.74 (18)	C15—C10—C11—C12	−1.4 (3)
O3—N5—C3—C4	5.9 (3)	C15—C10—C11—C16	178.6 (2)
C6—C1—C2—C3	0.0 (3)	N4—C10—C15—C14	177.10 (18)
C8—C1—C2—C3	−178.49 (19)	C11—C10—C15—C14	1.6 (3)
C2—C1—C6—N1	−179.38 (17)	C10—C11—C12—C13	0.6 (3)
C2—C1—C6—C5	0.1 (3)	C16—C11—C12—C13	−179.4 (2)
C8—C1—C6—N1	−0.5 (2)	C11—C12—C13—C14	0.0 (3)
C8—C1—C6—C5	178.98 (18)	C12—C13—C14—C15	0.2 (3)
C2—C1—C8—N2	−3.3 (3)	C13—C14—C15—C10	−1.0 (3)
C2—C1—C8—C7	178.38 (19)		

### Hydrogen-bond geometry (Å, °)

**Table:**

*D*—H···*A*	*D*—H	H···*A*	*D*···*A*	*D*—H···*A*
N3—H3···O1	0.86	2.04	2.7074 (17)	134
N4—H4A···N2	0.86	2.20	2.6254 (18)	110
N1—H1···O4^i^	0.86	2.02	2.8394 (19)	160
O4—H4B···S1^ii^	0.82	2.55	3.3485 (14)	164
C16—H16C···O3^iii^	0.96	2.45	3.342 (3)	154

Symmetry codes: (i) *x*, −*y*+1/2, *z*+1/2; (ii) −*x*+1, −*y*, −*z*+1; (iii) *x*, −*y*+3/2, *z*−1/2.
